# MiR-1202 increases radioresistance in nasopharyngeal carcinoma by targeting NAIF1/MAPK/ERK pathway

**DOI:** 10.1016/j.isci.2026.115135

**Published:** 2026-02-25

**Authors:** Xuxia Chen, Youqin Du, Weiling Chen, Xiaohui Yang, Jingwei Fang, Song Qu

**Affiliations:** 1 Department of Radiation Oncology, Guangxi Medical University Cancer Hospital, Nanning, China; 2 Key Laboratory of Early Prevention and Treatment for Regional High Frequency Tumor (Guangxi Medical University), Ministry of Education, Nanning, China; 3 Guangxi Medical University, Nanning, China

**Keywords:** Biochemistry, Cancer, Cancer systems biology, Molecular biology, Therapy

## Abstract

Radiotherapy is a primary treatment for nasopharyngeal carcinoma (NPC), yet intrinsic and acquired radioresistance limits therapeutic efficacy. Here, we investigated the functional role and mechanism of miR-1202 in NPC radioresponse. Using *in vitro* cell models and *in vivo* xenografts, we show that miR-1202 overexpression enhances cell survival, clonogenic capacity, and tumor growth following ionizing radiation (IR), whereas miR-1202 suppression produces the opposite effects. Mechanistically, miR-1202 directly targets NAIF1, leading to activation of the MAPK/ERK signaling pathway and modulation of apoptosis-related proteins, characterized by increased Bcl-2 and decreased BAX expression under IR. Notably, miR-1202 regulates ERK1/2 phosphorylation without affecting total ERK1/2 levels, indicating post-translational control of pathway activation. These findings identify a miR-1202-NAIF1-MAPK/ERK regulatory axis that contributes to NPC radioresistance and highlight miR-1202 as a potential biomarker and therapeutic target to improve radiotherapy outcomes.

## Highlights

• miR-1202 contributes to radioresistance of NPC *in vitro* and *in vivo*
• miR-1202 enhances NPC radioresistance by directly targeting NAIF1 and activating MAPK/ERK
• miR-1202 confers radioprotection in NPC by suppressing IR-induced apoptosis

## Graphical abstract

**Figure:**
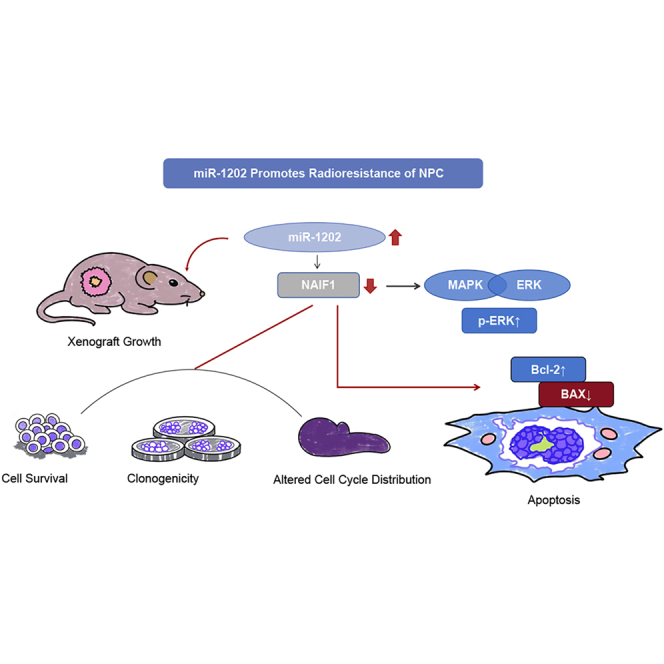

## Introduction

Nasopharyngeal carcinoma (NPC) is a malignant tumor originating from the epithelium of the nasopharynx, most commonly arising in the pharyngeal recess. According to global cancer statistics, approximately 133,000 new cases of NPC were diagnosed in 2020, accounting for only 0.7% of all newly diagnosed cancers worldwide.^1^ Although the global incidence of NPC is relatively low, its geographical distribution is highly uneven, with 70% of new cases occurring in East and Southeast Asia.^2^ This distinctive pattern renders NPC a significant public health concern in these regions. Due to its unique anatomical location and high sensitivity to ionizing radiation (IR), radiotherapy has become the primary treatment modality for non-metastatic NPC.^3^ In recent years, the development of intensity-modulated radiotherapy (IMRT) has significantly improved local control rates, thereby enhancing the prognosis of patients with NPC.^4^ However, in routine clinical practice, we have observed that some patients with NPC fail to achieve favorable therapeutic outcomes despite receiving standard treatment. Studies have reported that approximately 20% of patients still develop local recurrence and distant metastasis after therapy.^5^^,^^6^ Such variability is attributable to factors including tumor histology, stage of disease, patient age, and general physical status. Moreover, inter-individual differences in radiosensitivity represent a key determinant of treatment response. Radioresistance has emerged as a major obstacle to the efficacy of radiotherapy in NPC patients.^7^^,^^8^ Nevertheless, to date, the molecular mechanisms underlying radioresistance in NPC remain poorly understood.

MicroRNAs (miRNAs) are a class of endogenous, evolutionarily conserved, non-coding small RNAs with a length of 19–25 nucleotides. They exert their biological functions by binding to the 3′ untranslated region (3′UTR) of target gene mRNAs, thereby mediating post-transcriptional gene silencing.^9^^,^^10^^,^^11^ Dysregulation of miRNAs is closely associated with tumor initiation and progression, and they hold significant clinical value in cancer diagnosis, prognosis prediction, and therapeutic intervention.^12^ Previous studies have shown that miRNAs can participate in the regulation of key cellular processes involved in the response to IR, such as apoptosis, proliferation, DNA damage repair, and angiogenesis, thereby influencing tumor radiosensitivity.^13^^,^^14^ Increasing evidence has indicated a close association between miRNAs and radiosensitivity in NPC.^15^^,^^16^^,^^17^ In our previous study, a microarray-based screening of serum samples from radioresistant and radiosensitive patients with NPC identified 37 differentially expressed miRNAs. Among them, miR-1202 was significantly upregulated in the serum of radioresistant NPC patients.^18^

miR-1202 is a primate-specific miRNA.^19^ A growing body of evidence has implicated it in tumorigenesis and cancer progression. However, its expression pattern and functional roles vary across different cancer types. For instance, miR-1202 is upregulated and exhibits oncogenic properties in endometrial cancer, adrenocortical carcinoma, papillary thyroid carcinoma, and gastric cancer.^20^^,^^21^^,^^22^^,^^23^ Conversely, it is downregulated in hepatocellular carcinoma, cervical cancer, glioma, lung cancer, and clear cell papillary renal cell carcinoma, and has been shown to function as a tumor suppressor.^24^^,^^25^^,^^26^^,^^27^^,^^28^ To date, the expression profile and functional significance of miR-1202 in NPC remain largely unexplored. Moreover, no studies have yet reported on the specific role of miR-1202 in mediating radioresistance in malignant tumors, including NPC.

Nuclear apoptosis-inducing factor 1 (NAIF1) is ubiquitously expressed in human tissues and is evolutionarily conserved across species. Overexpression of NAIF1 has been shown to inhibit cell proliferation and promote apoptosis.^29^ Prior studies have revealed that NAIF1 expression is frequently downregulated in various types of cancer. For example, in gastric cancer, NAIF1 downregulation is associated with enhanced cell proliferation, altered cell cycle progression, and increased invasion and metastasis.^30^^,^^31^^,^^32^ A study on non-small cell lung cancer (NSCLC) indicated that NAIF1 can suppress cell proliferation and is correlated with overall survival in NSCLC patients.^33^ Furthermore, overexpression of NAIF1 has been shown to inhibit both proliferation and invasiveness in osteosarcoma^34^ and prostate cancer cells.^35^ Our previous study demonstrates that overexpression of NAIF1 enhances the radiosensitivity of CNE-2R cells.^36^ In this study, we further confirmed a direct targeting relationship between miR-1202 and NAIF1 and investigated their roles in modulating the radioresponse of NPC.

The mitogen-activated protein kinase (MAPK) signaling pathway is a classical signal transduction cascade in eukaryotic cells. It includes four main subtypes: extracellular signal-regulated kinases (ERK), p38, c-Jun N-terminal kinases (JNK), and ERK5. Aberrant ERK activation is involved in approximately one-third of all human cancers.^37^^,^^38^ Studies have shown that IR can activate ERK in tumor cells, and activated ERK confers protection to cancer cells by inhibiting apoptosis, promoting DNA repair, and inducing cell-cycle arrest, thereby mitigating the cytotoxic effects of IR.^39^

The present study aimed to investigate the role and underlying molecular mechanisms of the miR-1202-NAIF1-MAPK/ERK axis in regulating radiosensitivity in NPC. We found that miR-1202 enhances radioresistance in NPC both *in vitro* and *in vivo*. Mechanistically, miR-1202 directly targets NAIF1, leading to activation of the MAPK/ERK signaling pathway and subsequent enhancement of radioresistance. Furthermore, we demonstrated that miR-1202 upregulates the anti-apoptotic protein Bcl-2 and downregulates the pro-apoptotic protein BAX in NPC cells following IR, thereby exerting a radioprotective effect.

In summary, this study elucidates the critical role of miR-1202 in mediating radioresistance in NPC. Therapeutic targeting of miR-1202 may represent a promising approach to enhance the radiosensitivity of NPC. These findings provide a theoretical framework for developing miR-1202 as a potential molecular biomarker of NPC radiosensitivity and suggest its relevance for radiosensitization-based therapeutic strategies.

## Results

### miR-1202 is highly expressed in radioresistant NPC cell line CNE-2R

**Figure 1:**
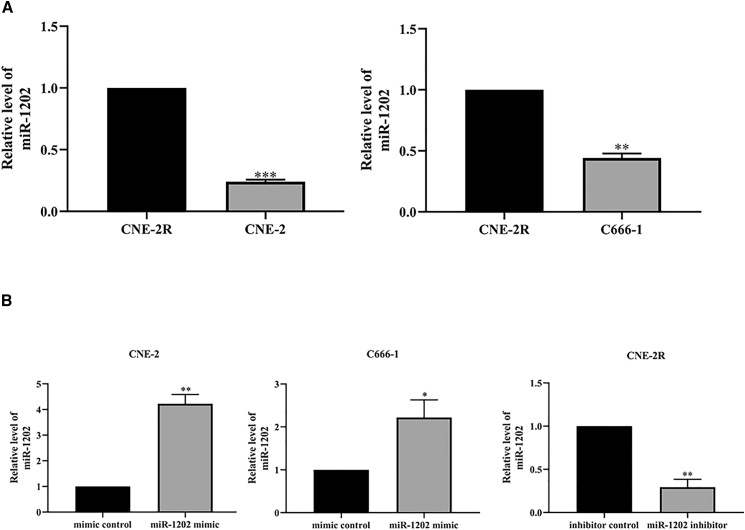
miR-1202 overexpression is associated with a radioresistant phenotype of NPC *in vitro* (A) The expression of miR-1202 in CNE-2, C666-1, and CNE-2R cells was detected by RT-qPCR. The U6 gene was used as housekeeping gene. Samples were normalized to the CNE-2R cultures. (B) CNE-2 and C666-1 cell lines were transfected with either a control vector (mimic control) or a miR-1202 overexpression vector (miR-1202 mimic). CNE-2R cell line was transfected with either a control vector (inhibitor control) or a miR-1202 silence vector (miR-1202 inhibitor). RT-qPCR was used to measure miR-1202 expression post-transfection. The U6 gene was used as housekeeping gene for RT-qPCR analysis (mean ± SD; *n* = 3; *t* test; ∗*p* < 0.05, ∗∗*p* < 0.01, ∗∗∗*p* < 0. 001, ∗∗∗∗*p* < 0. 0001).

In our previous clinical study, we observed elevated serum levels of miR-1202 in radioresistant NPC patients compared to radiosensitive counterparts. To further explore its role, we examined miR-1202 expression in NPC cell lines. RT-qPCR analysis revealed significantly higher expression of miR-1202 in the radioresistant cell line CNE-2R than in its parental line CNE-2. Moreover, when compared with another NPC cell line, C666-1, CNE-2R also exhibited markedly increased miR-1202 expression (Figure 1A), indicating a potential association between miR-1202 expression and radioresistance in NPC.

### miR-1202 enhances radioresistance in NPC cells

**Figure 2:**
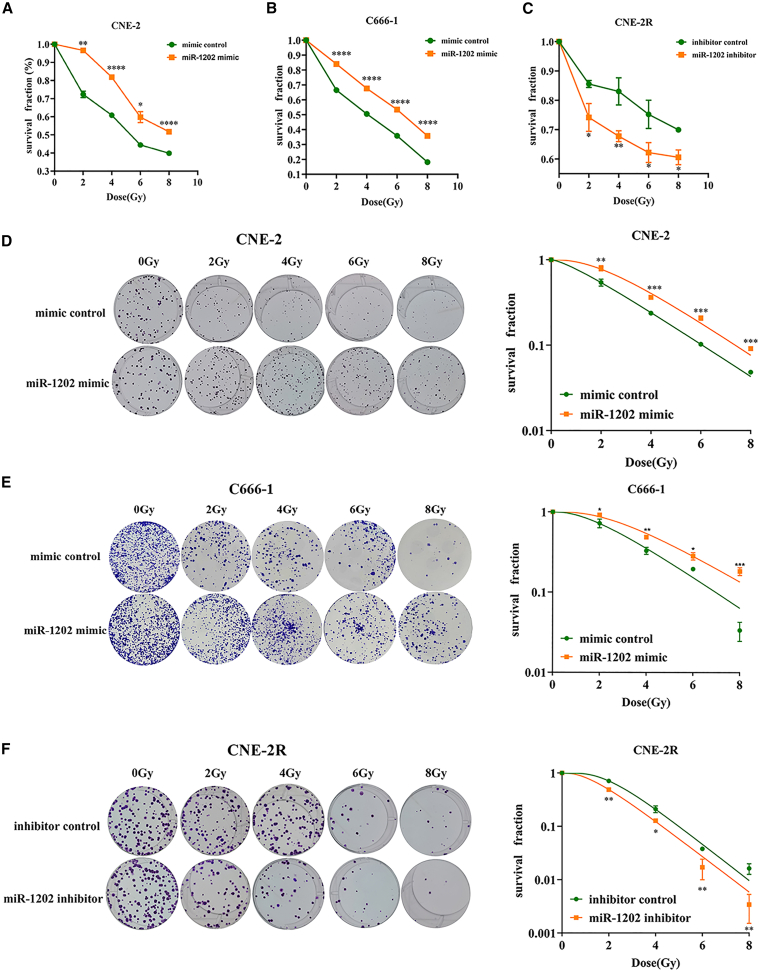
MiR-1202 enhances radioresistance of NPC *in vitro* (A–C) The survival fraction of miR-1202 mimic-transfected CNE-2 (A), miR-1202 mimic-transfected C666-1 (B), miR-1202 inhibitor-transfected CNE-2R (C) cells and their control cells after 0, 2, 4, 6, and 8 Gy of X-ray IR was measured by CCK-8 assay; (D–F) The colony formation ability of miR-1202 mimic-transfected CNE-2 (D), miR-1202 mimic-transfected C666-1 (E), miR-1202 inhibitor-transfected CNE-2R (F) cells and their control cells after 0, 2, 4, 6, and 8 Gy of X-ray IR was measured by colony-forming assay and the dose-survival curves were calculated and fitted to a single-hit multi-target model (mean ± SD; *n* = 3; *t* test; ∗*p* < 0.05, ∗∗*p* < 0.01, ∗∗∗*p* < 0.001, ∗∗∗∗*p* < 0.0001).

To investigate the functional role of miR-1202 in radiosensitivity of NPC, we established stable cell lines overexpressing miR-1202 in CNE-2 and C666-1, and silenced miR-1202 in CNE-2R (Figure 1B). CCK-8 assays showed that upon exposure to X-ray IR (0–8 Gy), miR-1202-overexpressing CNE-2 and C666-1 cells exhibited higher survival fractions, whereas miR-1202-knockdown reduced survival in CNE-2R, compared with their respective control cells (Figures 2A–2C).

Colony formation assays demonstrated that miR-1202 overexpression significantly enhanced clonogenic survival in CNE-2 and C666-1 following 0–8 Gy X-ray IR compared to control cells, while its inhibition reduced colony formation in CNE-2R. Survival curves, fitted using the single-hit multi-target model, confirmed that miR-1202 conferred radioresistance to NPC cells (Figures 2D–2F).

**Figure 3:**
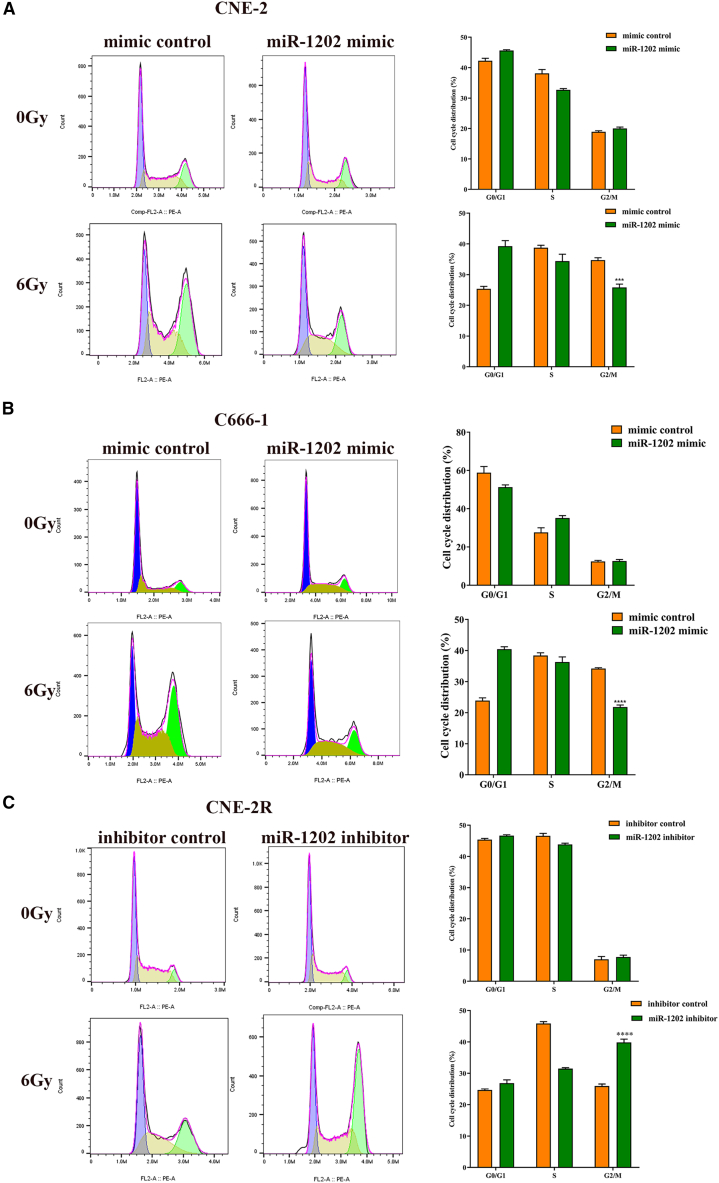
MiR-1202 enhances radioresistance of NPC *in vitro* (A–C) The cell cycle distribution of miR-1202 mimic-transfected CNE-2 (A), miR-1202 mimic-transfected C666-1 (B), miR-1202 inhibitor-transfected CNE-2R (C) cells and their control cells after 0 or 6 Gy of X-ray IR was detected by flow cytometry (mean ± SD; *n* = 3; *t* test; ∗*p* < 0.05, ∗∗*p* < 0.01, ∗∗∗*p* < 0.001, ∗∗∗∗*p* < 0.0001).

Cell cycle analysis revealed that under non-irradiated conditions, miR-1202 had minimal impact on cell cycle distribution. However, following 6 Gy X-ray IR, miR-1202-overexpressing CNE-2 and C666-1 cells showed a decreased proportion of G2/M phase cells compared to control cells, while knockdown of miR-1202 in CNE-2R increased G2/M arrest (Figures 3A–3C). These data suggest that miR-1202 enhances radioresistance of NPC cell by promoting survival and modulating cell cycle progression after IR exposure.

### NAIF1 is a direct target of miR-1202

**Figure 4:**
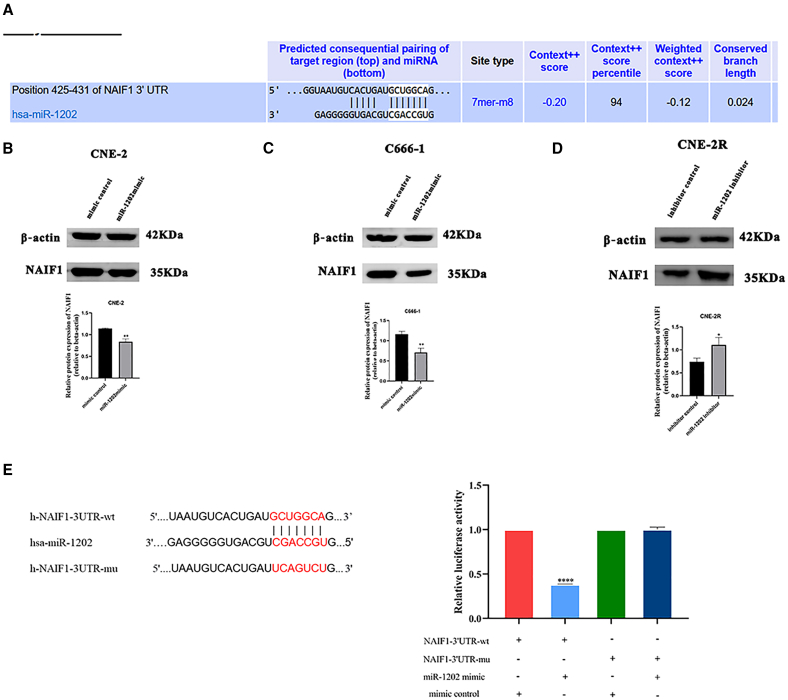
NAIF1 is a direct target of miR-1202 (A) The binding site between miR-1202 and the 3′ UTR of NAIF1 was predicted by Targetscan. (B–D) The protein expression of NAIF1 in miR-1202-overexpression and -knockdown NPC cells and their control cells was detected by Western blot. (E) Relative luciferase activity of 293T cells co-transfected with PSI-Check2-NAIF1-3′UTR-wt or PSI-Check2-NAIF1-3′URT-mu reporter plasmids and mimic control or miR-1202 mimic (mean ± SD; *n* = 3; *t* test; ∗*p* < 0.05, ∗∗*p* < 0.01, ∗∗∗*p* < 0.001, ∗∗∗∗*p* < 0.0001).

To identify downstream effectors of miR-1202, bioinformatics analysis using TargetScan (http://www.targetscan.org), miRDB (http://www.mirdb.org), and miRWalk (http://mirwalk.umm.uni-heidelberg.de/) predicted NAIF1 as a candidate target gene. A potential miR-1202 binding site was identified within the 3′-untranslated region (3′ UTR) of NAIF1 (Figure 4A). Western blot analysis revealed that miR-1202 overexpression reduced NAIF1 protein levels in CNE-2 and C666-1, whereas miR-1202 knockdown upregulated NAIF1 in CNE-2R compared with their respective control cells (Figures 4B–4D).

A dual-luciferase reporter assay confirmed direct binding of miR-1202 to the NAIF1 3′ UTR. miR-1202 significantly suppressed relative luciferase activity of the wild-type NAIF1 3′ UTR construct, but had no effect on the mutant construct lacking the predicted binding site (Figure 4E). These findings establish NAIF1 as a direct downstream target of miR-1202 in NPC.

### NAIF1 sensitizes NPC cells to IR

**Figure 5:**
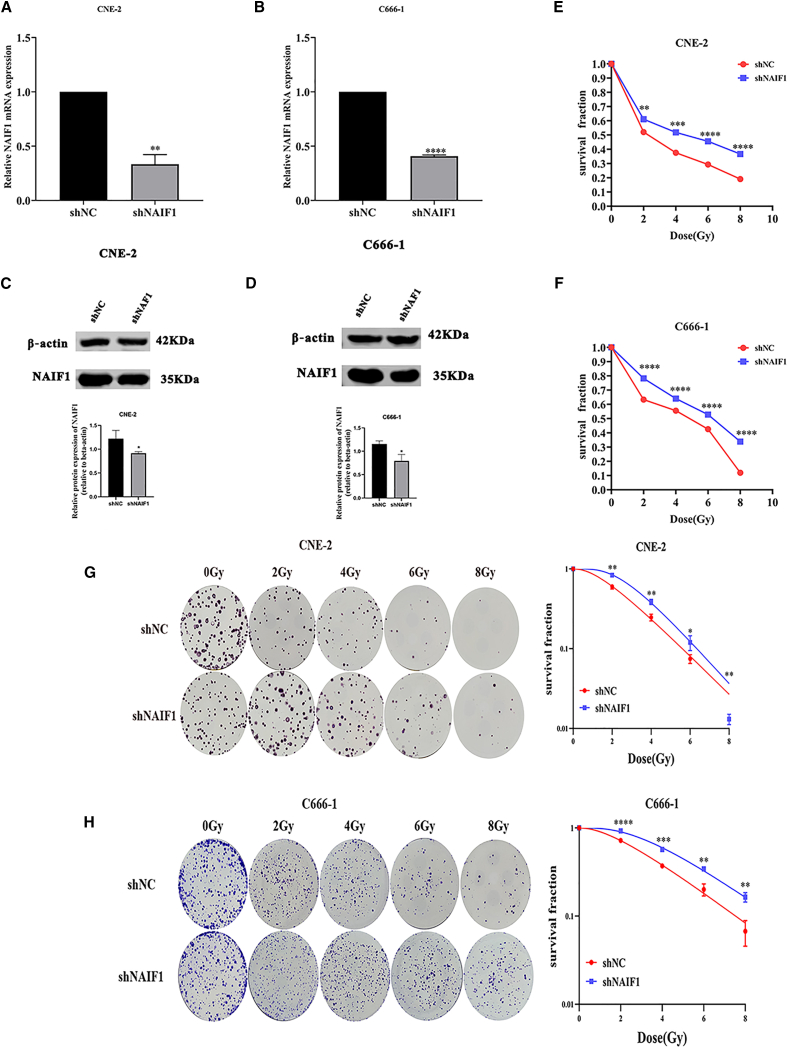
NAIF1 increases radiosensitivity of NPC *in vitro* (A and B) The mRNA expression of NAIF1 in CNE-2 and C666-1 cells after transfection was detected by RT-qPCR. The GAPDH gene was used as housekeeping gene. (C and D) The protein expression of NAIF1 in CNE-2 and C666-1 cells after transfection was detected by Western blot. (E and F) The survival fraction of NAIF1-knockdown CNE-2 and C666-1 cells and their control cells after 0, 2, 4, 6, and 8 Gy of X-ray IR was measured by CCK-8 assay. (G and H) The colony formation ability of the NAIF1-knockdown CNE-2 and C666-1 cells and their control cells after 0, 2, 4, 6, and 8 Gy of X-ray IR was measured by colony-forming assay; and the dose-survival curves were calculated and fitted to a single-hit multi-target model (mean ± SD; *n* = 3; *t* test; ∗*p* < 0.05, ∗∗*p* < 0.01, ∗∗∗*p* < 0.001, ∗∗∗∗*p* < 0.0001).

**Figure 6:**
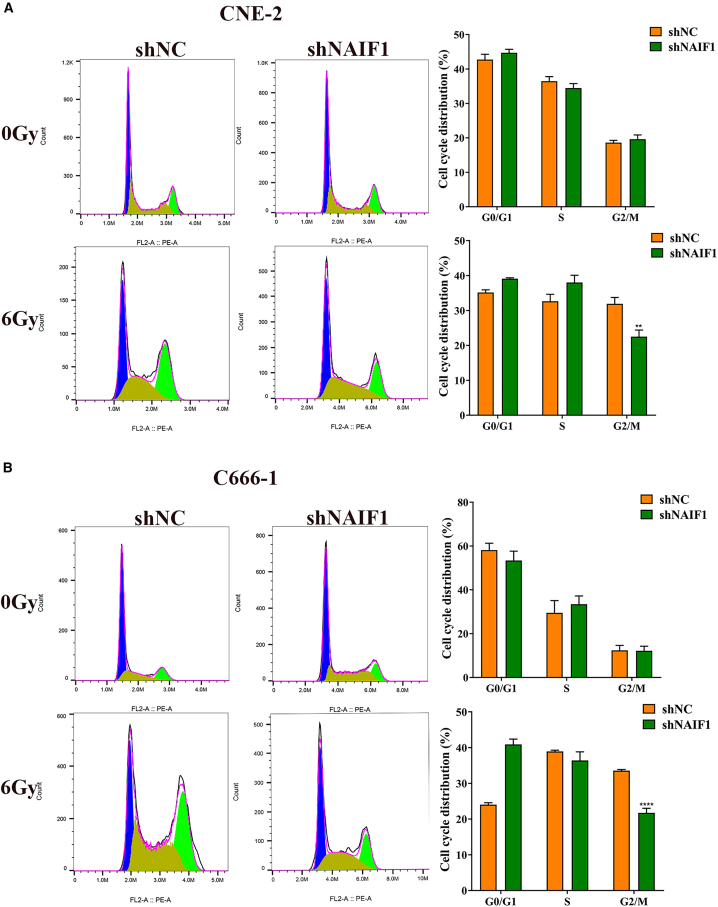
NAIF1 increases radiosensitivity of NPC *in vitro* (A and B) The cell cycle distribution of the NAIF1-knockdown CNE-2 and C666-1 cells and their control cells after 0 or 6 Gy of X-ray IR was detected by flow cytometry (mean ± SD; *n* = 3; *t* test; ∗*p* < 0.05, ∗∗*p* < 0.01, ∗∗∗*p* < 0.001, ∗∗∗∗*p* < 0.0001.

As demonstrated in our previous study, overexpression of NAIF1 significantly increased the radiosensitivity of CNE-2R cells. To further elucidate the role of NAIF1 in radiosensitivity of NPC, we silenced NAIF1 in CNE-2 and C666-1 cells (Figures 5A–5D). CCK-8 and colony formation assays demonstrated that NAIF1 knockdown increased cell survival and clonogenic formation in CNE-2 and C666-1 compared to controls (Figures 5E–5H). Cell cycle analysis revealed that NAIF1 knockdown inhibited G2/M arrest in CNE-2 and C666-1 (Figures 6A and 6B). These results suggest that NAIF1 enhances radiosensitivity of NPC *in vitro*.

### miR-1202 inhibits IR-induced apoptosis in NPC cells by targeting NAIF1

**Figure 7:**
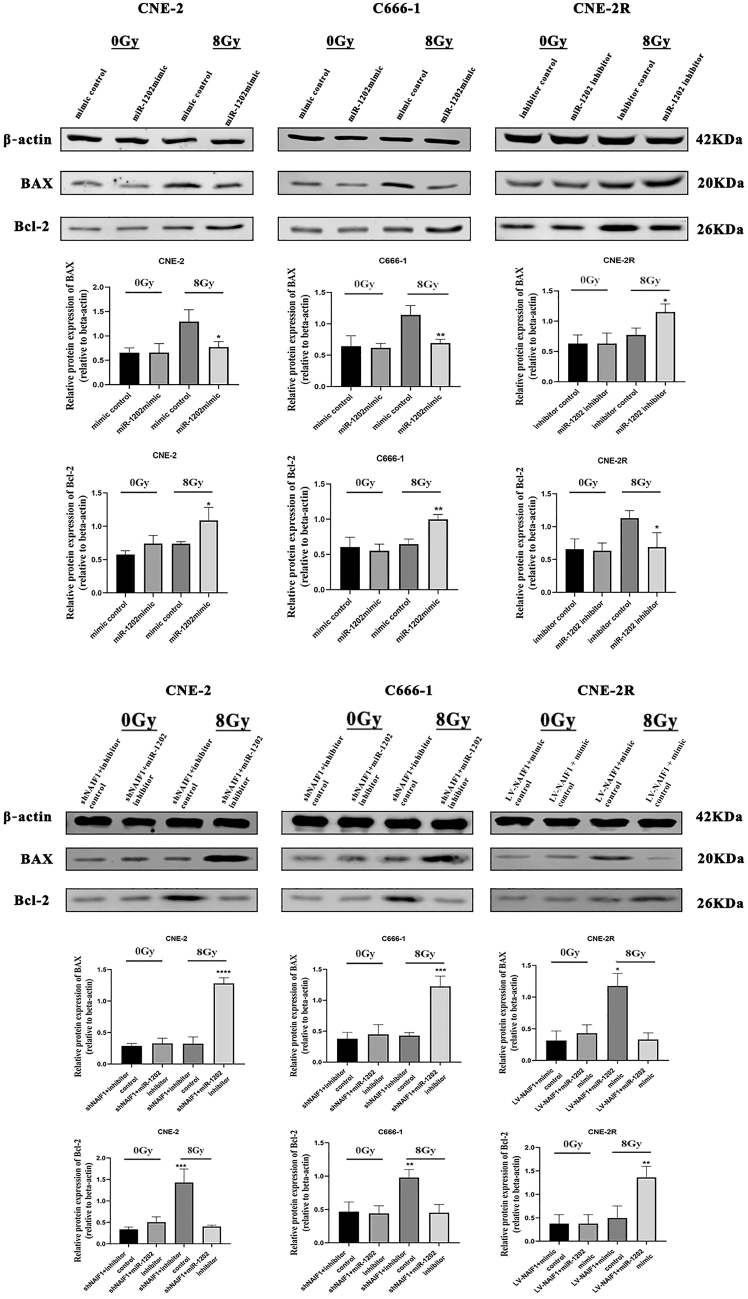
miR-1202 inhibits IR-induced apoptosis in NPC cells by targeting NAIF1 The expression of BAX and Bcl-2 protein in NPC cells and their control cells was measured by Western blotting after 0 or 8 Gy of IR (mean ± SD; *n* = 3; *t* test; ∗*p* < 0.05, ∗∗*p* < 0.01, ∗∗∗*p* < 0.001, ∗∗∗∗*p* < 0.0001).

To determine whether miR-1202 influences apoptosis in response to IR, we assessed the expression of the pro-apoptotic protein BAX and anti-apoptotic protein Bcl-2 by Western blotting. Under non-IR conditions, miR-1202 had negligible effects on BAX or Bcl-2 expression. However, following 8 Gy X-ray IR, BAX expression was suppressed and Bcl-2 expression was increased in miR-1202-overexpressing CNE-2 and C666-1 cells compared to control. Conversely, miR-1202 knockdown in CNE-2R resulted in increased BAX and decreased Bcl-2 levels compared to control. Moreover, under IR conditions, silencing miR-1202 reversed the effects of NAIF1 knockdown on apoptosis-related proteins, restoring the expression of the pro-apoptotic protein BAX and reducing the level of the anti-apoptotic protein Bcl-2, whereas miR-1202 overexpression produced the opposite effects (Figure 7). These results indicate that miR-1202 contributes to radioresistance of NPC by attenuating IR-induced apoptosis through targeting NAIF1.

### miR-1202 promotes radioresistance of NPC by targeting NAIF1

**Figure 8:**
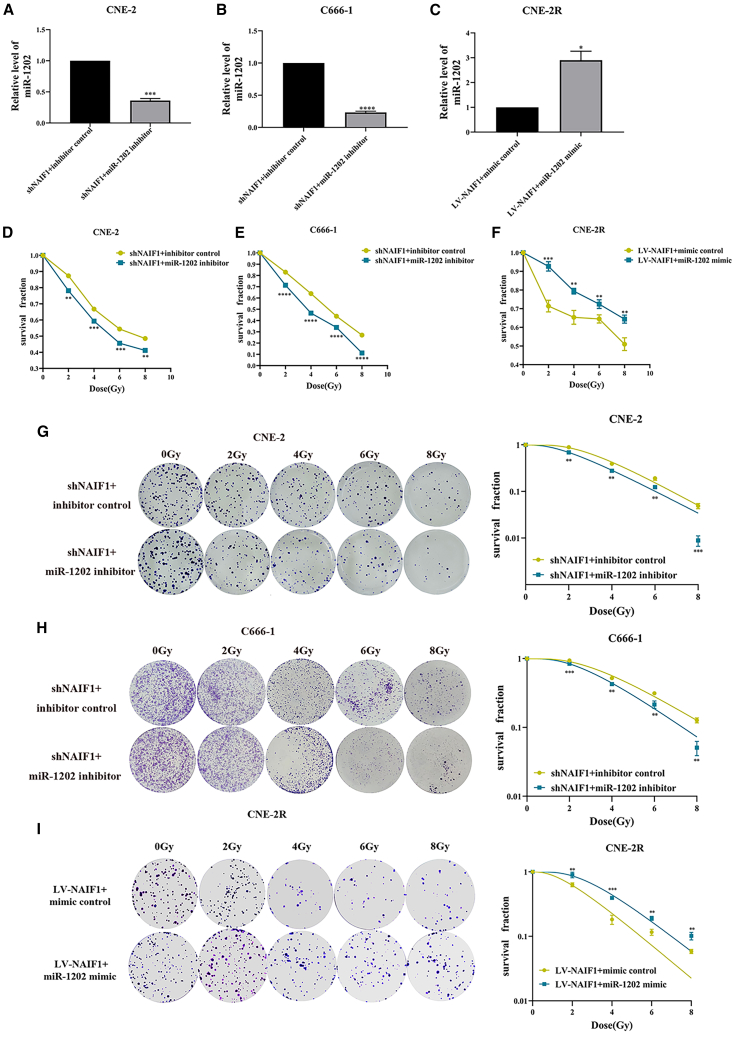
MiR-1202 increases radioresistance of NPC by targeting NAIF1 (A–C) NAIF1-knockdown CNE-2 and C666-1 cells were transfected either a control vector (inhibitor control) or a miR-1202 silence vector (miR-1202 inhibitor); NAIF1-overexpression CNE-2R cells were transfected either a control vector (mimic control) or a miR-1202 overexpression vector (miR-1202 mimic). (D–F) The survival fraction of cotransfected NPC cells after 0, 2, 4, 6, and 8 Gy of X-ray IR was detected by CCK-8 assay. (G–I) The colony formation ability of cotransfected NPC cells after 0, 2, 4, 6, 8 Gy of X-ray IR was detected by colony-forming assay and the dose-survival curves were calculated and fitted to a single-hit multi-target model (mean ± SD; *n* = 3; *t* test; ∗*p* < 0.05, ∗∗*p* < 0.01, ∗∗∗*p* < 0.001, ∗∗∗∗*p* < 0.0001).

**Figure 9:**
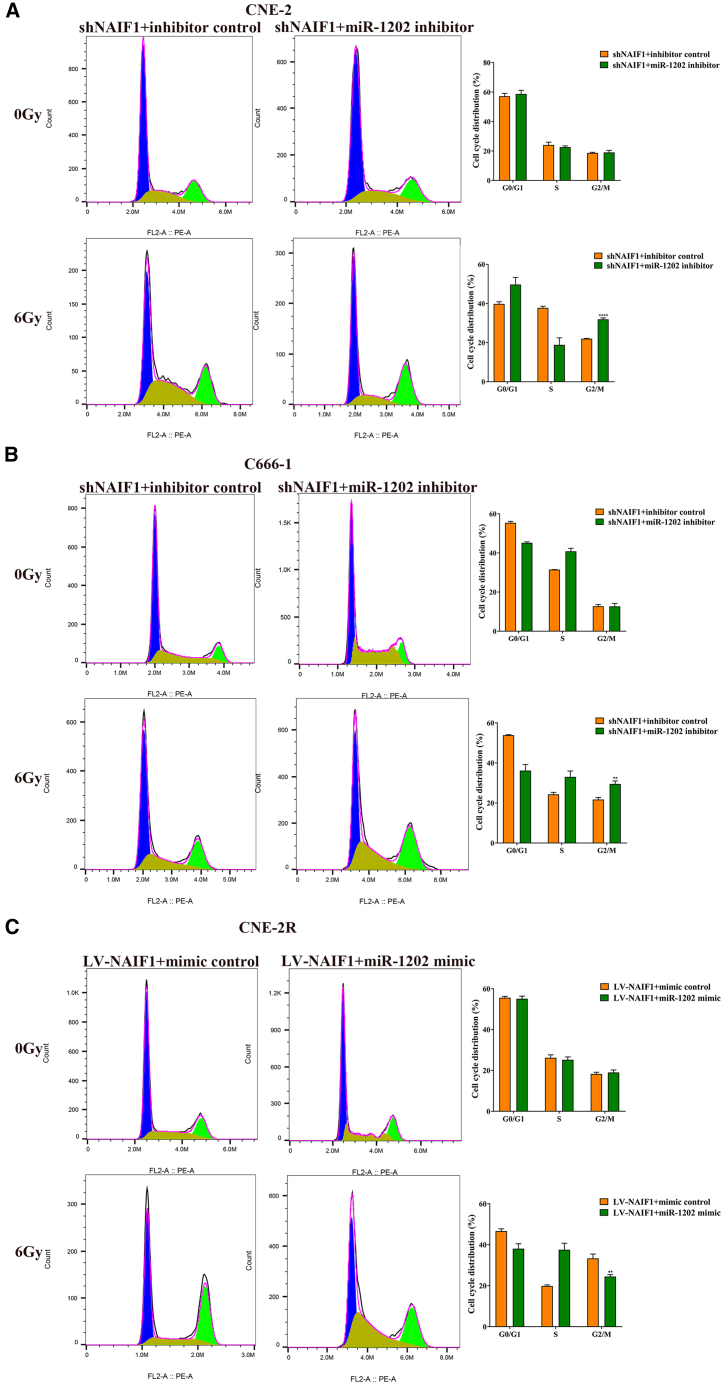
MiR-1202 increases radioresistance of NPC by targeting NAIF1 (A–C) The cell cycle distribution of cotransfected NPC cells after 0 or 6 Gy of X-ray IR was detected by flow cytometry (mean ± SD; *n* = 3; *t* test; ∗*p* < 0.05, ∗∗*p* < 0.01, ∗∗∗*p* < 0.001, ∗∗∗∗*p* < 0.0001).

To verify whether miR-1202 can participate in the regulation of radiosensitivity in NPC cells by targeting NAIF1, corresponding vectors were co-transfected into NPC cells to conduct the rescue experiments (Figures 8A–8C). We validated that overexpression of miR-1202 effectively rescued the inhibitory effects of NAIF1 upregulation on cell survival and colony formation of NPC post-IR and that miR-1202 inhibition exerted the opposite effect (Figures 8D–8I). Moreover, miR-1202 overexpression reversed NAIF1-induced G2/M arrest in CNE-2R cells, while miR-1202 inhibition restored the decrease G2/M phase cell population caused by NAIF1 silencing (Figures 9A–9C). Collectively, these findings confirm that miR-1202 promotes radioresistance of NPC through targeting NAIF1.

### miR-1202 promotes IR-induced activation of the MAPK/ERK pathway by targeting NAIF1

**Figure 10:**
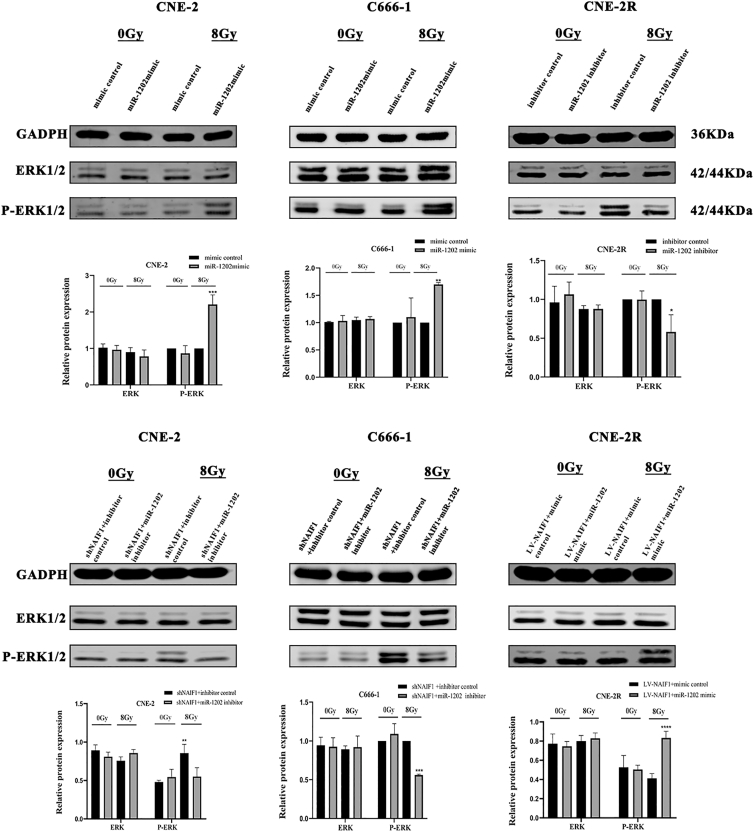
MiR-1202 promotes IR-induced MAPK/ERK activation by targeting NAIF1 The expression of ERK1/2 and p-ERK1/2 in NPC cells was measured by Western blotting after 0 or 8 Gy of IR. (mean ± SD; *n* = 3; *t* test; ∗*p* < 0.05, ∗∗*p* < 0.01, ∗∗∗*p* < 0.001, ∗∗∗∗*p* < 0.0001).

Previous studies have demonstrated the radioprotective role of the MAPK/ERK pathway in various tumors, and evidence suggests that NAIF1 functions as a negative regulator of MAPK/ERK signaling.^30^ Therefore, we investigated whether the miR-1202-NAIF1 axis enhances radioresistance of NPC by modulating the activity of the MAPK/ERK signaling pathway. As show in Figure 10 under both IR and non-IR conditions, overexpression of miR-1202 in CNE-2 and C666-1 cells and silencing of miR-1202 in CNE-2R cells did not significantly affect the protein levels of total ERK1/2. In addition, in the established NPC cell line models, neither overexpression nor silencing of miR-1202 induced significant changes in phosphorylated ERK1/2 (p-ERK1/2) without IR compared with their respective control groups. However, following 8 Gy IR, overexpression of miR-1202 markedly increased the protein levels of phosphorylated ERK1/2 (p-ERK1/2) in CNE-2 and C666-1 cells, while miR-1202 knockdown significantly suppressed p-ERK1/2 expression in CNE-2R cells, compared with their respective controls. Furthermore, after 8 Gy irradiation, miR-1202 downregulation reversed the enhanced ERK1/2 phosphorylation induced by NAIF1 silencing in CNE-2 and C666-1 cells, whereas miR-1202 overexpression abrogated the suppressive effect of NAIF1 overexpression on ERK phosphorylation in CNE-2R. These results suggest that under IR stress, miR-1202 promotes IR-induced MAPK/ERK activation by targeting NAIF1.

### miR-1202 enhances radioresistance of NPC *in vivo*

**Figure 11:**
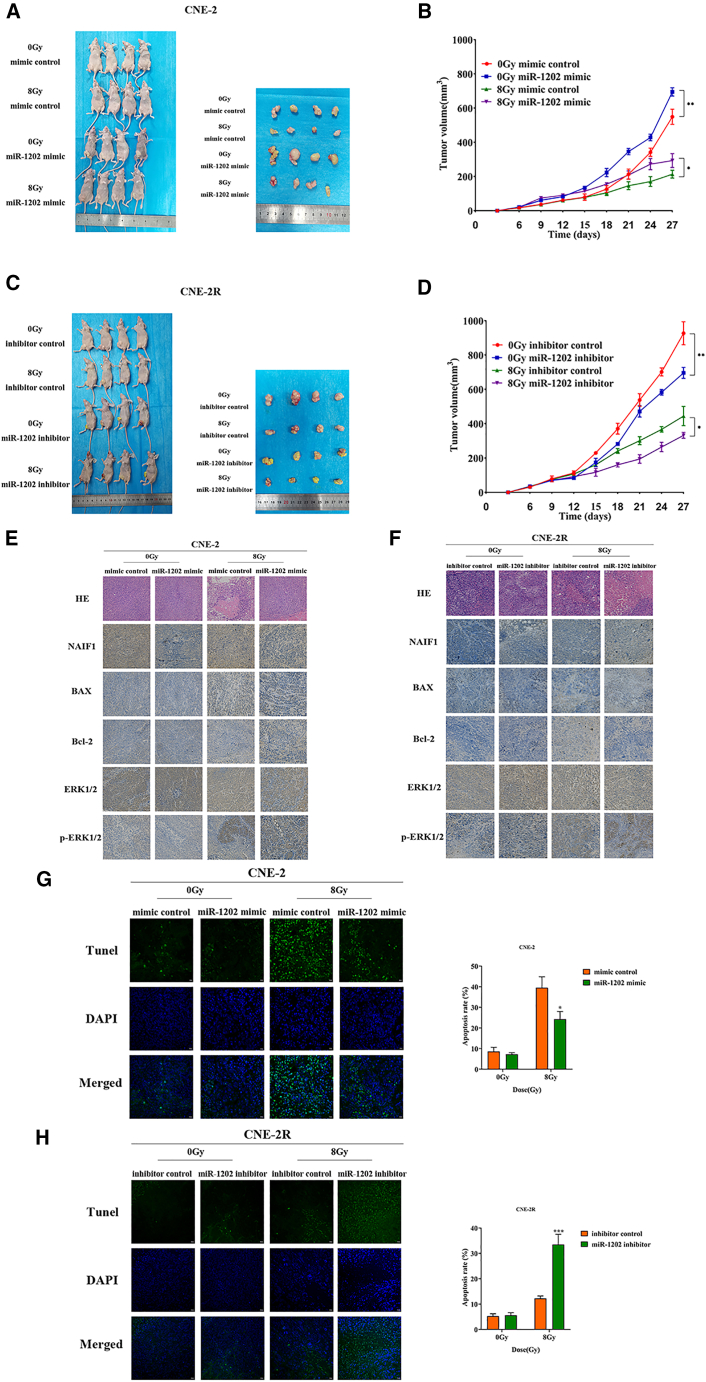
MiR-1202 increases radioresistance of NPC *in vivo* (A and B) The gross appearance and volume of NPC xenograft of miR-1202-overexpression CNE-2 cells and their control cells with or without 8 Gy of X-ray IR. (C and D) The gross appearance and volume of NPC xenograft of miR-1202-knockdown CNE-2R with or without 8 Gy of X-ray IR. (E) Representative picture of HE staining and NAIF1, BAX, Bcl-2, ERK1/2, and p-ERK1/2 immunostaining in NPC xenografts of the mimic control and miR-1202 mimic with or without 8 Gy of X-ray IR (200×). (F) Representative picture of HE staining and NAIF1, BAX and Bcl-2, ERK1/2, and p-ERK1/2 immunostaining in NPC xenografts of the inhibitor control and miR-1202 inhibitor with or without 8 Gy of X-ray IR (200×). (G) Representative images of TUNEL staining in NPC xenografts of the mimic control and miR-1202 mimic, the apoptosis rate was calculated as the percentage of TUNEL positive cells. Scale bars, 20 μm (400×). (H) Representative images of TUNEL staining in NPC xenografts of inhibitor control and miR-1202 inhibitor, the apoptosis rate was calculated as the percentage of TUNEL positive cells. Scale bars, 20 μm (400×) (mean ± SD; *n* = 3; *t* test; ∗*p* < 0.05, ∗∗*p* < 0.01, ∗∗∗*p* < 0.001, ∗∗∗∗*p* < 0.0001).

To validate the role of miR-1202 *in vivo*, we established subcutaneous xenografts in nude mice using miR-1202-perturbated CNE-2, CNE-2R cells as well as the respective control cells. Tumor-bearing mice were treated with or without 8 Gy X-ray IR. Tumor growth was monitored over time. In the absence of IR, miR-1202 overexpression promoted NPC xenograft growth, while silencing miR-1202 suppressed it, following 8Gy IR, NPC xenograft growth was suppressed in all groups, but this effect was attenuated in miR-1202-overexpressing CNE-2 tumors and enhanced in miR-1202-silenced tumors (Figures 11A–11D).

Subsequently, we performed immunohistochemistry (IHC) and TUNEL assays. First, we observed a consistent inverse correlation between miR-1202 expression and NAIF1 protein levels in NPC xenografts, further supporting the notion that NAIF1 is a direct target of miR-1202 in regulating radiosensitivity in NPC. Second, IHC results indicated that under non-IR conditions, miR-1202 expression had no significant effect on the levels of the apoptosis-related proteins BAX and Bcl-2. However, following exposure to 8 Gy of X-ray IR, miR-1202 overexpression enhanced the expression of the anti-apoptotic protein Bcl-2 and suppressed that of the pro-apoptotic protein BAX compared to control. Conversely, miR-1202 silencing increased BAX expression while reducing Bcl-2 expression. Similarly, under IR, miR-1202-silenced NPC xenografts exhibited a marked accumulation of TUNEL-positive nuclei, indicating enhanced apoptosis, whereas xenografts with overexpressing miR-1202 displayed a reduced number of apoptotic cells. These findings indicate that miR-1202 enhances radioresistance *in vivo* by inhibiting IR-induced apoptosis in NPC. Additionally, the data showed that miR-1202 overexpression significantly promoted ERK1/2 phosphorylation following 8 Gy of X-ray IR, while miR-1202 silencing produced the opposite effect (Figures 11E–11H).

Taken together, these *in vivo* data are consistent with our *in vitro* results, indicating that miR-1202 also plays a critical role in enhancing radioresistance of NPC *in vivo*.

### miR-1202/NAIF1-mediated radioresistance is dependent on the activation of the MAPK/ERK

**Figure 12:**
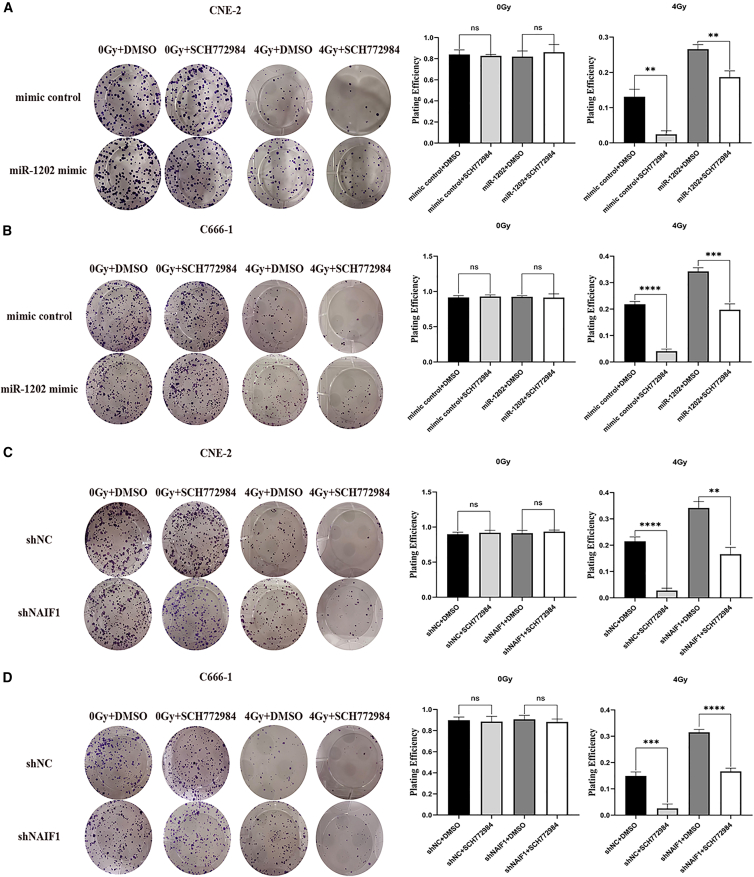
miR-1202/NAIF1 mediated radioresistance is dependent on the activation of the MAPK/ERK (A and B) Representative images of cell colonies and the corresponding quantification of plating efficiency of miR-1202 overexpressing and control NPC cell cultures with SCH772984 followed by 4 Gy of IR and then cultured for 14 days (C and D) Representative images of cell colonies and the corresponding quantification of plating efficiency of NAIF1 knockdown and control NPC cell cultures with SCH772984 followed by 4 Gy of IR and then cultured for 14 days (mean ± SD; *n* = 3; *t* test; ∗*p* < 0.05, ∗∗*p* < 0.01, ∗∗∗*p* < 0.001, ∗∗∗∗*p* < 0.0001).

To determine whether activation of the MAPK/ERK pathway is critically involved in miR-1202 overexpression- or NAIF1 silencing-induced radioresistance, we pharmacologically blocked MAPK/ERK activation and assessed clonogenic survival in cells with miR-1202 overexpression or NAIF1 knockdown under 0 Gy and 4 Gy IR. As shown in Figure 12, we observed that, in the absence of IR, inhibition of MAPK/ERK activation did not exert a noticeable impact on the clonogenic capacity of NPC cells. In contrast, after exposure to 4 Gy IR, suppression of ERK phosphorylation effectively reversed the radioresistant phenotype induced by miR-1202 overexpression or NAIF1 silencing. Moreover, in the control group—characterized by inherently low miR-1202 and high NAIF1 expression—treatment with an ERK inhibitor post-IR resulted in the lowest clonogenic survival, consistent with the most pronounced inhibition of ERK phosphorylation. Collectively, these findings indicate that MAPK/ERK activation is indispensable for miR-1202/NAIF1-mediated radioresistance in NPC cells.

## Discussion

This study systematically demonstrates the pivotal role of miR-1202 in modulating radioresistance in NPC. Building upon our previous findings that miR-1202 is significantly upregulated in the serum of radioresistant NPC patients, we further elucidated its functional significance and underlying mechanisms in radioresistance of NPC. Functionally, miR-1202 overexpression promoted NPC cells survival, clonogenic ability, and xenograft tumor growth, while reducing the proportion of cells in the G2/M phase upon IR. In contrast, knockdown of miR-1202 inhibited these phenotypes. Mechanistically, miR-1202 directly targets NAIF1, thereby activating the MAPK/ERK signaling pathway and enhancing radioresistance of NPC. Additionally, under IR conditions, miR-1202 exerts a radioprotective effect by promoting the expression of the anti-apoptotic protein Bcl-2 and suppressing the pro-apoptotic protein BAX in NPC.

Although no prior studies have addressed the role of miR-1202 in the tumor response to IR, its oncogenic functions have been reported in several cancers, including endometrial carcinoma, gastric cancer, thyroid carcinoma, and adrenocortical carcinoma.^20^^,^^21^^,^^22^^,^^23^ Conversely, miR-1202 functions as a tumor suppressor in other cancer types, such as endometrial cancer, hepatocellular carcinoma, lung cancer, glioma, and breast cancer,^24^^,^^25^^,^^26^^,^^27^^,^^28^ suggesting that its biological role is tumor type-dependent. Therefore, elucidating the regulatory mechanisms of miR-1202 within specific tumor is of great significance.

Alterations in apoptosis regulation are not only associated with tumor progression but also contribute to resistance to therapy.^40^ The intrinsic apoptotic pathway is primarily governed by proteins of the B-cell lymphoma 2 (Bcl-2) family.^41^ Under IR-induced stress, the intrinsic (mitochondria-dependent) apoptotic pathway can be activated, leading to programmed cell death.^42^ Malignant cells may evade apoptosis by disrupting the balance of Bcl-2 family proteins.^43^ In our study, we demonstrated that miR-1202 regulates the expression of Bcl-2 family proteins under IR. Specifically, miR-1202 overexpression led to upregulation of the anti-apoptotic protein Bcl-2 and downregulation of the pro-apoptotic protein BAX, thereby inhibiting the intrinsic apoptotic cascade. While modulation of miR-1202 levels was able to reverse the effects of NAIF1 overexpression or knockdown on BAX and Bcl-2 expression. In addition, previous studies have reported that NAIF1 can induce apoptosis.^29^ These findings collectively support the conclusion that modulation of apoptosis is a key mechanism by which miR-1202 confers radioresistance in NPC, providing a molecular basis for its radioprotective function.

The MAPK/ERK signaling pathway plays a critical role in regulating cellular responses to IR.^44^ Activated ERK participates in the modulation of tumor cell radiosensitivity through its involvement in cell proliferation and survival pathways.^45^ Growing evidence supports the radioprotective role of ERK activation in various malignancies, including rhabdomyosarcoma,^46^^,^^47^ prostate cancer,^48^ NPC,^49^^,^^50^ lung cancer,^51^^,^^52^^,^^53^ osteosarcoma,^54^ cervical cancer,^55^ glioma,^56^ and colorectal cancer.^57^ Given that miR-1202 enhances radioresistance in both *in vitro* and *in vivo* NPC models, we propose that miR-1202 may exert radioprotective effects through activation of the MAPK/ERK pathway in NPC. Notably, our study revealed that miR-1202 overexpression or silencing selectively modulates ERK1/2 phosphorylation without altering total ERK1/2 protein levels after IR, suggesting that miR-1202 regulates the MAPK/ERK pathway at the post-translational rather than transcriptional level. In addition to its direct influence on MAPK/ERK pathway components, miR-1202 may also act by modulating upstream regulators of this signaling cascade. Although we currently lack experimental evidence to confirm this hypothesis, further investigation will be undertaken in future studies.

Our results highlight translational potential. Circulating miRNAs are emerging as clinically informative biomarkers,^58^^,^^59^^,^^60^ and serum-based detection of miR-1202 may offer a minimally invasive approach for predicting radiosensitivity in NPC. Furthermore, given its functional role in promoting radioresistance, therapeutic inhibition of miR-1202 using antagomiRs or nanocarrier-based delivery systems represents a promising strategy to enhance radiotherapy efficacy.

### Limitations of the study

The study has several limitations that warrant further investigation. First, validation in additional radioresistant NPC cell lines is required to improve the generalizability of the proposed mechanism. Second, although elevated serum miR-1202 levels in radioresistant NPC patients support clinical relevance, validation in tumor tissues was limited by specimen availability and ethical constraints; future multicenter studies may help address this issue. Importantly, while our data support NAIF1 as a functional target of miR-1202, rescue experiments involving modulation of NAIF1 expression levels are still required to consolidate the upstream-downstream relationship between miR-1202 and NAIF1. Finally, prospective clinical studies are necessary to determine whether miR-1202 can serve as a reliable predictor of radiotherapy response.

## Resource availability

### Lead contact

Further information and requests for resources and reagents should be directed to and will be fulfilled by the lead contact, Song Qu (qusong2022@126.com).

### Materials availability

This study did not generate new unique reagents.

### Data and code availability

• All data reported in this article will be shared by the lead contact upon request.
• This article does not report original code.
• Any additional information required to reanalyze the data reported in this article is available from the lead contact upon request.

## Acknowledgments

This work was supported by the 10.13039/501100001809National Natural Science Foundation of China (10.13039/501100001809NSFC) [grant 82460536]; the Guangxi Natural Science Foundation [grant 2024GXNSFAA010398]; the Key Laboratory of Early Prevention and Treatment for Regional High Frequency Tumor (10.13039/501100011827Guangxi Medical University), Ministry of Education [grants GKE-ZZ202411 and GKE-ZZ202506]; and Guangxi Key Technologies R&D Program [grant GUIKEAB25069065]. We also thank Guangxi Medical University Cancer Hospital, Key Laboratory of Early Prevention and Treatment for Regional High Frequency for providing experimental facilities and technical support.

## Author contributions

X.C., writing – review and editing, formal analysis, and investigation; Y.D. and W.C., investigation and writing – original draft; X.Y., methodology and investigation; J.F., investigation; S.Q., conceptualization, funding acquisition, investigation, and supervision.

## Declaration of interests

The authors declare no conflicts of interest.

## STAR★Methods

### Key resources table

**Table:** 

REAGENT or RESOURCE	SOURCE	IDENTIFIER
**Antibodies**		

anti-NAIF1	Invitrogen	Cat# PA5-112979
anti-BAX	Cell Signaling Technology	Cat# 5023S; RRID: AB_10557411
anti-Bcl-2	Cell Signaling Technology	Cat# 15071S; RRID: AB_2744528
anti-ERK1/2	Cell Signaling Technology	Cat# 4695S; RRID: AB_390779
anti-phospho-ERK1/2	Cell Signaling Technology	Cat# 4376S; RRID: AB_331772
anti-β-actin	Cell Signaling Technology	Cat# 4970S; RRID: AB_2223172
anti-GADPH	Cell Signaling Technology	Cat# 5174S; RRID: AB_10622025
Goat anti-Rabbit IgG (H+L)	Invitrogen	Cat# ZG395294
Goat anti-Mouse IgG (H+L)	Invitrogen	Cat# ZJ401083

**Chemicals, peptides, and recombinant proteins**		

RIPA buffer	Beyotime	P0013B
RPMI1640 medium	Gibco	C11875500BT
Fetal Bovine Serum	Biological Industries	04001
Penicillin-streptomycin mixture	Solarbio	P1400-100
TRIzol reagent	TAKARA	9108
TBST Buffer	Sangon Biotech	C520009

**Critical commercial assays**		

miRNA first-strand cDNA synthesis (tailing) kit	Sangon Biotech	B5324651-0020
First Strand cDNA Synthesis Master Mix reagent kit	Sangon Biotech	B639251-0100
miRNA quantitative PCR kit	Sangon Biotech	B532461-0002
2xSG Fast qPCR Master Mix	Sangon Biotech	B639271-0005
CCK-8 kit	CELLCOOK	CT01A
PI/RNase Staining Buffer	Becton Dickinson Biosciences	550825
Enhanced BCA Protein Assay Kit	Beyotime	P0010
Dual-Luciferase Reporter Assay Kit	Hanbio	HB-DLR-100
Tunel Cell Apoptosis Detection Kit	Servicebio	G1504-50T

**Experimental models: Cell lines**		

Human:CNE-2	the experimental center of Fudan University Shanghai Cance Center	–
Human:CNE-2R	Guangxi Medical University	–
Human:C666-1	Guangxi Medical University	–

**Experimental models: Organisms/strains**		

BALB/C nude mice(female)	the Experimental Animal Center of Guangxi Medical University	–

**Oligonucleotides**		

hsa-miR-1202 inhibitor, sequence (5′-3′): CTCCCCCACTGCAGCTGGCAC	Genepharma	–
shRNA targeting sequence for NAIF1(5′-3′): GGCCTATGATCAAAGATTTCC,	Genechem	–
Primers for RT-qPCR, see Table S1	Sangon Biotech	–

**Software and algorithms**		

GraphPad Prism 8	GraphPad	–
SPSS 17.0	IBM	–

### Experimental model and study participant details

#### Experimental animals

To generate a xenograft NPC model, 4-week-old female BALB/C nude mice were obtained from the Experimental Animal Center of Guangxi Medical University, randomly divided into four groups, and raised in specific pathogen-free (SPF) conditions. Only female mice were used, and sex-related effects were not evaluated in this study. All animal experiments were approved by the Ethics Committee of Guangxi Medical University cancer Hosptitol (KY2025611).

#### Cell culture

The NPC cell line CNE-2 used in this study was obtained from the experimental center of Fudan University Shanghai Cance Center. The NPC cell line C666-1 was provided and maintained by Guangxi Medical University. Radiation-resistant NPC cell line CNE-2R was constructed and preserved by our laboratory. The cell lines CNE-2 ,C666-1 and CNE-2R, were cultured in RPMI1640 medium (Gibco, USA) containing 10% fetal bovine serum and 1% penicillin-streptomycin mixture. All cells were cultured in a 37°C incubator with 5% CO2.

### Method details

#### Cell lines, cell culture, and cell irradiation

The NPC cell line CNE-2 used in this study was obtained from the experimental center of Fudan University Shanghai Cance Center. The NPC cell line C666-1 was provided and maintained by Guangxi Medical University. Radiation-resistant NPC cell line CNE-2R was constructed and preserved by our laboratory. The parental cell lines CNE-2 and C666-1 were authenticated by short tandem repeat (STR) profiling prior to use and all cell lines were routinely tested for mycoplasma contamination. The cell lines CNE-2, C666-1 and CNE-2R, were cultured in RPMI1640 medium (Gibco, USA) containing 10% fetal bovine serum and 1% penicillin-streptomycin mixture. All cells were cultured in a 37°C incubator with 5% CO2. The cells were irradiated with 6MV X-ray generated by a linear accelerator at a dose rate of 400 cGy/min.

#### Reverse transcription-quantitative PCR (RT-qPCR)

Reverse transcription-quantitative PCR (RT-qPCR) was used to analyze the expression of miR-1202 and NAIF1. Total RNA was extracted using TRIzol reagent (Takara, Japan) and reverse transcribed into cDNA using miRNA first-strand cDNA synthesis (tailing) kit (Sangon Biotech, China) or MightyScript First Strand cDNA Synthesis Master Mix reagent kit (Sangon Biotech, China). RT-qPCR was performed using a miRNA quantitative PCR kit (Sangon Biotech, China) or 2xSG Fast qPCR Master Mix (Sango Biotech, China). The relative expression level of miR-1202 was normalized against U6 using the 2^-ΔΔCt^ method. The relative expression level of NAIF1 was normalized against GAPDH using the 2^-ΔΔCt^ method. The primers for miR-1202, NAIF1 and U6 were designed and synthesized by Sangon Biotech (Shanghai) Co., Ltd. The primer sequences can be found in Table S1.

#### Cell transfection

CNE-2, C666-1 and CNE-2R cells in their logarithmic growth phase were seeded in 6-well plates at 5x10^4^ cells/well. Upon adhesion to the walls, cells were transfected with the lentiviral vectors expressing either miR-1202, NAIF1, or the negative controls with a multiplicity of infection (MOI) of 30. An inverted fluorescence microscope (Thermo, USA) assessed the transfection efficiency.

#### Cell counting kit (CCK-8) assay

Cells of each group were seeded into 96-well plates at 2 x 10^3^ cells/well in triplicates and were either exposed or not exposed to different doses of radiation according to the requirement of the experiment. Then each well was incubated with 10ul CCK-8 solution (CELLCOOK, China) for 2 h. Subsequently, the absorbance (OD) value of cells at 450 nm was measured by a microplate reader (Thermo, USA).

#### Flow cytometry analysis

PI/RNase Staining Buffer (Becton Dickinson Biosciences, USA) was used to detect the cycle distribution of cells exposed to 0Gy or 6Gy IR. Briefly, cells were collected after receiving 0Gy or 6Gy of X-ray IR and fixed overnight with 75% ethanol solution at 4°C. The BD FACS Calibur flow cytometer was used to detect cell cycle distribution using the PI-stained cells.

#### Colony-forming assay

Cells were seeded in 6-well plates (each cell line with each exposure dose had three replicate wells). After 12 h, the cells were irradiated with 0, 2, 4, 6, and 8 Gy of IR. After irradiation, cells were cultured in a 37°C incubator with 5% CO2 for 14 days. Subsequently, the colonies were fixed with 4% paraformaldehyde fix solution and stained with Giemsa staining solution. Then, the number of colonies was counted using a microscope (A colony was defined as having ≥50 cells). The following formulas were used to calculate the Plating Efficiency (PE) and Survival Fraction (SF) under each dose of irradiation: Plating Efficiency (PE) = the number of cell colonies/number of seeded cells×100%; Survival Fraction (SF) = PE_nGy_/PE_0Gy_.

#### Western blotting

Proteins were extracted from cells using RIPA buffer (Beyotime, China), and the concentration was measured by the BCA method. An equal amount of protein in each sample was separated on 10% SDS-PAGE and blotted on the PVDF membrane (Biosharp, China). After blocking with 5% non-fat milk, blots were washed with TBST and incubated with primary antibodies overnight at 4°C, including anti-NAIF1 (1:1000, Invitrogen, USA), anti-BAX (1:1000, CST, USA), anti-Bcl-2(1:1000, CST, USA), anti-ERK1/2 (1:1000, CST, USA), anti-phospho-ERK1/2 (1:1000, CST, USA), anti-β-actin(1:1000, CST, USA), anti-GADPH(1:1000, CST, USA), followed by incubation with Goat anti-Rabbit IgG (H+L) Dylight 800 4X PEG(1:15000, Invitrogen, USA) or Goat anti-Mouse IgG (H+L) Dylight 800 4X PEG (1:15000, Invitrogen, USA) for 1h at room temperature. The protein bands were detected by an Odyssey CLx Imaging system and analyzed with ImageJ software.

#### Dual-luciferase reporter assay

To verify whether miR-1202 directly interacts with NAIF1, a wild-type NAIF1 3′ URT reporter plasmid (NAIF1-3′ UTR-wt) and a mutant NAIF1 3′ URT reporter plasmid (NAIF1-3′ UTR-mut) was constructed, both plasmids containing a pGL3 promoter. 293T cells were co-transfected with PSI-Check2-NAIF1-3′UTR-wt or PSI-Check2-NAIF1-3′URT-mut reporter plasmids and miR-1202 mimic or mimic control plasmids. The firefly luciferase activity was normalized to Renilla luciferase activity.

#### Xenograft NPC model in nude mouse

4-week-old female BALB/C nude mice were used for *in vivo* studies.1.0×10^7^ cells were injected subcutaneously into the left groin of nude mice, and the xenograft growth was observed every 3 days. An electronic vernier caliper measured the long and short diameter of the tumor, while the tumor volume was calculated according to the formula: Tumor volume = 1/2 long diameter × short diameter. When the tumor volume reached about 80-120mm^3^ (about 21 days after cell injection), they were exposed to 0Gy or 8Gy irradiation. Anesthesia was induced in tumor-bearing nude mice by intraperitoneal injection of chloral hydrate (2.5 ml/kg). The tumor in the left groin of the nude mice was fully exposed to the linear accelerator irradiation field, and the rest of the body was covered with lead blocks. The irradiation was performed using x-rays with an energy of 6 MV at a dose rate of 600 monitor units (MU)/min (6Gy/min). Subsequently, the nude mice were killed by cervical dislocation 33 days after injection, the tumor was excised, the volume was measured, and the growth curve of the Xenograft tumor was generated. Animals that died unexpectedly during the experiment were excluded. Efforts were made to minimize the number of animals utilized and to decrease their suffering.

#### Immunohistochemistry

The xenograft tumor was extracted from the nude mice, fixed, embedded, paraffin sectioned, dehydrated, and washed with buffer solution. The endogenous peroxidase was removed with 3% hydrogen peroxide solution, followed by soaking in sodium citrate buffer at pH 6.0 and boiling in a pressure cooker for 30 minutes. After cooling, the tissue sections were incubated with primary antibody overnight at 4°C, followed by incubation with secondary antibody at room temperature for 1h. All samples were stained, dehydrated, rinsed, and sealed.

#### Terminal deoxynucleotidyl transferase-mediated dUTP nick and labeling (TUNEL) assay

TUNEL-positive cells in xenograft tumor tissues were quantified using a TissueFAXS cell analysis system (Tissue Genostics, Austria). The apoptosis index (AI) was calculated using the formula: AI=Tunel-positive cells/total cells × 100%.

### Quantification and statistical analysis

#### Statistical analysis

Data were analyzed using GraphPad Prism (version 8) SPSS (version 17.0). Statistical details for each experiment and the statistical tests applied, are provided in the corresponding figure legends.

Data are presented as the mean ± standard deviation (SD). Comparisons between two groups were performed using two-tailed Student’s t-tests, as indicated. Unless otherwise specified, all experiments were independently repeated at least three times. A P value < 0.05 was considered statistically significant.
