# Supplementary material for: MiR-1202 increases radioresistance in nasopharyngeal carcinoma by targeting NAIF1/MAPK/ERK pathway
Source: iScience. 2026 Feb 25;29(4):115135. doi: 10.1016/j.isci.2026.115135 (PMC13053778; doi:10.1016/j.isci.2026.115135)
Supplement: Document S1. Table S1 [file mmc1.pdf]

iScience, Volume *29*

Supplemental information

MiR-1202 increases radioresistance in nasopharyngeal carcinoma by targeting NAIF1/MAPK/ERK pathway

Xuxia Chen, Youqin Du, Weiling Chen, Xiaohui Yang, Jingwei Fang, and Song Qu

Supplementary Table 1: RT-qPCR primers used in this study

Genes	Forward primer (5’-3’)	Reverse primer (5’-3’)
NAIF1	5’-AGAGACCCCTGTGGACATGATGG-3’	5’-TTGGCGGAGTTGAGAGCAATGC-3’
miR-1202	5’-AGTGCCAGCTGCAGTGGG-3’	5’-TGAAGACACAGCAGATGACAGATATGG-3’
U6	5’-CGCGATATGGTTTTGGCAGG -3’	5’-TGGACGTATTCGATCAGCCG -3’
GAPDH	5’-CAGGAGGCATTGCTGATGAT-3’	5’-GAAGGCTGGGGCTCATTT-3’
